# What is the Optimal Duration of Middle-Cerebral Artery Occlusion Consistently Resulting in Isolated Cortical Selective Neuronal Loss in the Spontaneously Hypertensive Rat?

**DOI:** 10.3389/fneur.2015.00064

**Published:** 2015-03-26

**Authors:** Sohail Ejaz, David J. Williamson, Ulf Jensen-Kondering, Tahir Ahmed, Steve J. Sawiak, Jean-Claude Baron

**Affiliations:** ^1^Stroke Research Group, Department of Clinical Neurosciences, University of Cambridge, Cambridge, UK; ^2^Department of Clinical Neurosciences, Wolfson Brain Imaging Centre, Addenbrooke’s Hospital, University of Cambridge, Cambridge, UK; ^3^INSERM U 894, Université Paris Descartes, Paris, France

**Keywords:** stroke, focal cerebral ischemia, reperfusion, microglial activation, rat models

## Abstract

**Introduction and objectives:** Selective neuronal loss (SNL) in the reperfused penumbra may impact clinical recovery and is thus important to investigate. Brief proximal middle cerebral artery occlusion (MCAo) results in predominantly striatal SNL, yet cortical damage is more relevant given its behavioral implications and that thrombolytic therapy mainly rescues the cortex. Distal temporary MCAo (tMCAo) does target the cortex, but the optimal occlusion duration that results in isolated SNL has not been determined. In the present study, we assessed different distal tMCAo durations looking for consistently pure SNL.

**Methods:** Microclip distal tMCAo (md-tMCAo) was performed in ~6-month old male spontaneously hypertensive rats (SHRs). We previously reported that 45 min md-tMCAo in SHRs results in pan-necrosis in the majority of subjects. Accordingly, three shorter MCAo durations were investigated here in decremental succession, namely 30, 22, and 15 min (*n* = 3, 3, and 7 subjects, respectively). Recanalization was confirmed by MR angiography just prior to brain collection at 28 days and T2-weighted MRI was obtained for characterization of ischemic lesions. NeuN, OX42, and GFAP immunohistochemistry appraised changes in neurons, microglia, and astrocytes, respectively. Ischemic lesions were categorized into three main types: (1) pan-necrosis; (2) partial infarction; and (3) SNL.

**Results:** Pan-necrosis or partial infarction was present in all 30 min and 22 min subjects, but not in the 15 min group (*p* < 0.001), in which isolated cortical SNL was consistently present. MRI revealed characteristic hyperintense abnormalities in all rats with pan-necrosis or partial infarction, but no change in any 15 min subject.

**Conclusion:** We found that 15 min distal MCAo consistently resulted in pure cortical SNL, whereas durations equal or longer than 22 min consistently resulted in infarcts. This model may be of use to study the pathophysiology of cortical SNL and its prevention by appropriate interventions.

## Introduction

In acute stroke, the ischemic penumbra, i.e., the severely hypoxic and functionally impaired but still salvageable region surrounding the ischemic core, is the main target for reperfusion therapy (1, 2). Early reperfusion salvages penumbral tissue and reduces final infarct size and eventual disability in both animal models and human patients (3, 4).

Even though early reperfusion is clearly beneficial, the rescued penumbra may be affected by selective neuronal loss (SNL) (5, 6). SNL in the rescued penumbra following temporary middle cerebral artery occlusion (tMCAo) is particularly intriguing as it pertains to thrombolysis, an increasingly prevalent clinical scenario often resulting in substantial volumes of salvaged penumbra and commensurate neurological recovery. Although the functional impact of SNL in humans is still unclear (7–9), animal models have shown that it induces significant, albeit subtle, motor dysfunction lasting weeks (10). SNL is therefore a potential new therapeutic target and is an important phenomenon to investigate. Developing appropriate rodent models of salvaged penumbra is therefore of interest.

Reliable rodent models of striatal SNL using proximal MCAo and occlusion durations 15–30 min are available (10–14). These models can also induce associated cortical SNL, but this is inconsistent and mild at best (6, 10, 11, 13, 15). Yet, cortical damage is particularly relevant to clinical stroke given its long-lasting behavioral implications and that reperfusion therapy mainly targets the cortex. It is therefore important to try and develop a rodent model consistently resulting in cortical SNL not associated with an infarct, i.e., isolated or “pure” SNL.

Distal MCAO is a rodent stroke model that targets the cortex only, and is therefore appropriate for these purposes (16, 17). We previously reported that 45 min microclip distal temporary MCAo (md-tMCAo) in spontaneously hypertensive rats (SHRs), a strain clinically highly relevant, induces an infarct with associated SNL in the majority of subjects and pure SNL in a minority (18, 19). In the present study, we tested shorter md-tMCAo durations looking for an optimal duration consistently resulting in pure cortical SNL.

## Materials and Methods

The study was approved by the University of Cambridge Ethical Review Panel. In accordance with the legislation of UK Animals Scientific Procedures Act 1986 and The University of Cambridge Ethical Review Panel, it was designed so as to keep the number of animals used to a minimum, yet sufficient to obtain meaningful results. At 28 days after reperfusion, all rats were subjected to MRI scanning followed by perfusion–fixation of the brain for histological investigations.

For this study, we followed the ARRIVE guidelines for *in vivo* animal reports in stroke research (20), except that randomization between groups was not feasible given the specific aim of this investigation, namely to look for an MCAo duration that did not result in infarction in any subject yet consistently resulted in cortical SNL. Accordingly, a specific design was used, as follows. Given our already-mentioned previous studies showing the presence of cortical infarcts in several SHRs subjected to 45 min md-tMCAo (18, 19), we first studied the effects of 30 min MCAo in a batch of three rats studied simultaneously, then reduced the duration of MCAo in further batches of three rats until we found a duration leading to no infarct but consistent SNL. We then studied a further batch of four rats with the same MCAo duration to confirm the findings. In total, 13 young (~6 months) male SHRs of weight ~300 g (Charles River, UK) were used for the present study.

### Anesthesia

Anesthesia was induced with 4% isoflurane administered in a 0.3 l/min O_2_ and 0.7 l/min N_2_O mix and maintained with 2% isoflurane during surgical procedures. Body temperature was monitored with a rectal probe and maintained at 37.0 ± 0.5°C using a heated pad throughout all procedures. Moreover, continuous blood oxygen saturation and heart beat were monitored using a pulse-oximeter, and were within physiological ranges throughout all experiments.

### MCAo

Distal clip MCAo was performed using Buchan’s method (16) as implemented in our laboratory (18, 19, 21). Briefly, the left common carotid artery (CCA) was isolated through a ventral midline incision on the neck and a loose ligature of 4–0 silk suture was placed around it. With the rat positioned onto its right flank, a 2.5 cm skin incision perpendicular to and bisecting a line between the lateral canthus of the right eye and the external auditory canal was made, and the underlying temporalis muscle excised to reveal the base of the skull. Under direct visualization, the underlying temporalis muscle was excised and craniectomy was performed under saline irrigation to expose the left MCA through a 2-mm burr hole drilled 2–3 mm rostral to the fusion of the zygomatic arch with the squamosal bone. The dura was retracted to visualize the MCA at a position where it crosses the inferior cerebral vein, which lies within the rhinal fissure. A micro-aneurysm clip (No 1, Codman, Sundt AVM, USA) was placed on the MCA proximal to the point where it crosses the inferior cerebral vein in the rhinal fissure, and then the left CCA was permanently ligated. After the prescribed time (see above), the clip was removed and the wound was closed after visual verification of the restoration of blood flow (19). All animals were allowed to survive for 28 days.

### MRI acquisition

MRI was carried out immediately before perfusion-fixation at 28 ± 3 days of reperfusion, using the same anesthesia and physiological monitoring as with the surgery. Images were acquired using a 4.7T Bruker BioSpec 47/40 system (Bruker, BioSpin GmbH, Ettlingen, Germany) with a 2 cm surface coil used for signal reception. Since in our previous study using exactly the same MCAo protocol (save for the duration of 45 min), MR angiography (MRA) confirmed MCA recanalization in all rats (18), MRA was performed in only a subset of rats in the present study (*n* = 3, all from the 15 min MCAo group). Structural imaging was performed with a T2-weighted RARE sequence (TR/TE 3500/36 ms, ETL 8, slice thickness 1 mm, in plane resolution 0.156 mm). Images were assessed visually looking for abnormalities suggestive of an infarct (i.e., hypertense T2-weighted lesions) matching the histopathological lesions (see below). Quantitative lesion volumetry was not an aim of the present study.

### Perfusion-fixation

Immediately after completion of the MRI, the experimental protocol was terminated by intraperitoneal injection (30 mg/100 g) of sodium pentobarbitone. The animals were placed in supine position on a perfusion table and the thorax was opened through a midline incision. A dosing needle attached to a perfusion pump was inserted into the ascending aorta via the left ventricle and secured by silk suture. After incising the right atrium, cold (4°C) physiological saline (pH 7.4) was infused until the perfusate from the right atrium was clear (bloodless). They were then perfused with 100–150 ml of 1% gelatine solution followed by 150–200 ml of 4% paraformaldehyde (pH 7.4) solution. The animals were decapitated after perfusion and brains were quickly removed from the cranium and dissected free of the olfactory bulbs. Brains were post fixed in 4% paraformaldehyde overnight (4°C) and then immersed into 30% sucrose solution for cryoprotection.

### Post mortem procedures

#### Cryosectioning

The whole brain was embedded in OCT and mounted on specimen holder of a freezing sledge microtome for serial coronal sections (40 μm). Sections were collected across the MCA territory, i.e., from the level of the forceps minor of the corpus callosum to the visual cortex and the superior colliculi (Bregma +3.7 to -6.80 mm) (19) and mounted on gelatine coated slides.

#### Histopathology and immunohistochemistry

For immunohistochemistry (IHC), frozen brain sections were quenched with 10% methanol and 10% H_2_O_2_ for 5 min and blocked in 3% normal horse serum (S-2000, Vector Laboratories, Inc.). Consecutive sections were incubated overnight (ON) at 4°C with primary antibody specific for nuclear protein for mature neurons (NeuN, 1:1000; MAB377, Chemicon International), reactive microglia and macrophages (OX42, 1:400; MCA275R, Serotec Ltd.), and glial fibrillary acidic protein (GFAP, 1:500; G3893, Sigma-Aldrich Company Ltd.). After washing three times with TBS, sections were incubated with biotinylated anti-mouse IgG (rat-absorbed: dilution 1:200; Vector) secondary antibodies, washed with TBS, incubated in avidin–biotin horseradish peroxidase complex solution (ABC Kit, Vector Laboratories, Burlingame, CA, USA) for 10 min, and the reaction product visualized with diaminobenzidine (DAB Kit, Vector Laboratories, Burlingame, CA, USA) as the final chromogen. Finally, the sections were dehydrated through alcohol, cleared with xylene, and slides were cover-slipped with DPX mounting medium (1330-20-7, Merck).

Cresyl violet (CV) staining used a procedure adapted from previously published protocols (21). Briefly, frozen brain sections were dehydrated in 70, 95, and 100% ethanol for 15 min, and then immersed back through 95%, 70% ethanol and distilled water. The sections were stained with 0.5% CV for 30 min, then mounted in DPX media after dehydration and clearing.

#### Microscopic assessment of ischemic lesions

For the present study, we used the descriptive methodology based on visual identification and classification of ischemic lesion types detailed in our recent publication (18). It was outside the aim of this study to provide quantitative volumetry of lesions or actual cell-counting data, which is the topic of separate publications (22, 23). Briefly, based on classic literature definitions (11, 24), ischemic lesions were classified into three main subtypes, i.e., (1) pan-necrosis (tissue necrosis with absence of neurons, microglia, and astrocytes; dissolved extracellular matrix; cavitations and tissue loss); (2) partial infarction (same as pan-necrosis but only mild volume loss, relatively preserved extracellular matrix, no or few small cavitations, presence of dense activated microglia, and elongated astrocytes); and (3) SNL (patchy loss of neurons with preserved tissue structure and presence of activated microglia and elongated astrocytes matching SNL). The topography of ischemic changes was assessed for each rat according to our previously used cytoarchitectony-based template of regions-of-interest (ROIs) spanning the MCA territory across eight coronal sections extracted from the Paxinos and Watson Anatomic Atlas of the Rat Brain (25), located at bregmas +2.70, +1.00, -0.26, -0.92, -2.12, -3.14, -4.52, and -6.04 mm (18, 19, 26). As large cytoarchitectonic areas can span several coronal sections, the location of ischemic changes was recorded in terms of both (i) ROI affected; and (ii) sections, e.g., “primary somatosensory cortex (sections 2 and 3)” (see Table 1).

**Table 1:** **Summary of the histopathological and T_2_-weighted MR findings obtained in each rat 28 days after 15 min temporary MCA occlusion**.

tMCAo group	Subject	Histopathological findings^b^			T_2_-weighted MRI^c^
		Selective neuronal loss (SNL)	Partial infarction	Complete infarction	
30 min	1	+SI(1–3), SBF(5), M(1, 2, 5), I(2–4), V(8), A(8)	++ S2(3, 4, 6), SBF(4–6), I(2), A(7, 8), R(7)	++ S1(2), SBF(3–6), S2(4, 5), R(5, 7), A_7	++
30 min	2	+S1(1, 2, 6), S2(6), SBF(4, 5), A(7, 8), Pa(7), V(8)	++ S1(1, 2, 6), S2(3, 4), SBF(3–5), V(8)	++ Sl(2, 6), SBF(3–5), S2(3, 4), 1(2)	++
30 min	3	+ Sl(2, 6), S2(3), SBF(4–5), Ml(l), A(7)	++ M(1), S1(2, 3, 6), S2(3–5), SBF(4, 5), A(7), R(6, 7), V(8)	++ S1(2, 3, 6), SBF(3–6), S2(3–6), R(6), A(7), I(5), V(8)	++
22 min	1	+ S1(1, 3, 4, 6), SBF(4, 5), M(1, 2), 1(2, 3), V(8), A(7, 8), R(6), Pa(7)	++ Sl(l, 3), SBF(3–6), M(l, 2), I(2), S2(3–6), V(8), A(7, 8), Pa(7)	–	+
22 min	2^a^	–	–	–	–
22 min	3	+S1(1, 2, 3, 6), SBF(4, 5), I(2, 4), S2(3–5), A(8), R(6), Pa(8)	++ Sl(l, 2), SBF(3–6), S2(3–6), A(8), Pa(7)	–	+
15 min	1	+Sl(2), SBF(4–6), I(2–4), S2(4, 6), R(5, 6), Pa(5), V(8), A(7, 8)	–	–	–
15 min	2	+M(2), S1(2–6), SBF(3–6), I(2), S2(3–6), R(5–7), Pa(7), V(8), A(7, 8)	–	–	–
15 min	3	+ S1(2), I(3), R(5), V(8), A(8)	–	–	–
15 min	4	+ SBF(6), S2(6), R(6–8), A(7, 8)	–	–	–
15 min	5	+M(2, 3), S1(2, 4, 6), SBF(3, 4, 6), I(2, 4), S2(3, 4, 6), A(6)	–	–	–
15 min	6	+ S1(2–5), SBF(3–6), I(2, 4), P(2), C(2), S2(3–6), Pa(7), V(8), A(6, 8)	–	–	–
15 min	7	+ S1(5), SBF(3, 6), I(3, 5), S2(3, 5, 6), R(6–7), A(6)	–	–	–

*Code: + = moderate; ++ = severe; - = absent*.

*^a^This rat was found at post mortem to have unusual vascular anatomy of the MCA and no ischemic pathology and was excluded *post hoc* from further analysis*.

*^b^The location of the ischemic changes is listed according to the ROI affected and in brackets the sections where this ROI was affected (see Materials and Methods for details). Region code: A–auditory cortex, C–caudate putamen, I–insular cortex, M–motor cortex, P–piriform cortex, Pa–parietal cortex, R–rhinal cortex, S1–primary somatosensory cortex, S2–secondary somatosensory cortex, SBF–primary somatosensory cortex barrel field, T–thalamus, V–visual cortex. Section 1 = Bregma +2.70 mm; Section 2 = Bregma +1.00 mm; Section 3 = Bregma -0.26 mm; Section 4 = Bregma -0.92 mm; Section 5 = Bregma -2.12 mm; Section 6 = Bregma -3.14 mm; Section 7 = Bregma -4.52 mm; Section 8 = Bregma -6.04 mm*.

*^c^For SNL, no MRI changes could be identified*.

## Results

### Study timelines

All rats subjected to MCAo for this study are reported. No mortality was observed in any experimental group. As stated above, we first studied the effects of 30 min MCAo and then decreased the duration until we found a duration consistently resulting in pure SNL in three consecutive rats. As detailed below, all three rats subjected to 30 min MCAo exhibited partial or complete infarction. Accordingly, the occlusion time was reduced to 22 min. Using this occlusion duration, there was clear infarction in two rats; no tissue damage was found in the remaining subject probably in relation to an unusual MCA bifurcation noted on surgery, and this subject will not be described further. Based on these results, we further reduced the occlusion time to 15 min. This showed pure SNL in all three subjects. As per our planned design (see Materials and Methods), we therefore proceeded with an additional set of four rats with this duration. The detailed results in these three groups are detailed below.

### Histopathological results

Table 1 provides a detailed account of the histopathological findings in each rat for the three MCAo durations. Pan-necrosis or partial infarction, together with associated patchy SNL, were observed in all 30 min MCAo subjects; partial infarction and areas of SNL in both 22 min subjects; and isolated cortical SNL in all 15 min MCAo rats. Thus, all rats with either 30 or 22 min MCAo had infarction/partial infarction, whereas none of the 15 min MCAo rats did (*p* < 0.001, Fisher exact test), but all 15 min rats had cortical SNL, which was therefore isolated.

The topographic distribution of the ischemic damage is detailed in Table 1. The primary and secondary somatosensory areas (including the barrel fields), insular, and auditory cortices were most frequently affected, consistently across all three types of histopathological lesions. Note that Rat 6 of the 15 min group had SNL affecting the caudate nucleus (dorsal part), which in some SHRs belongs to the distal MCA vascular territory (16, 19).

**Figure 1:**
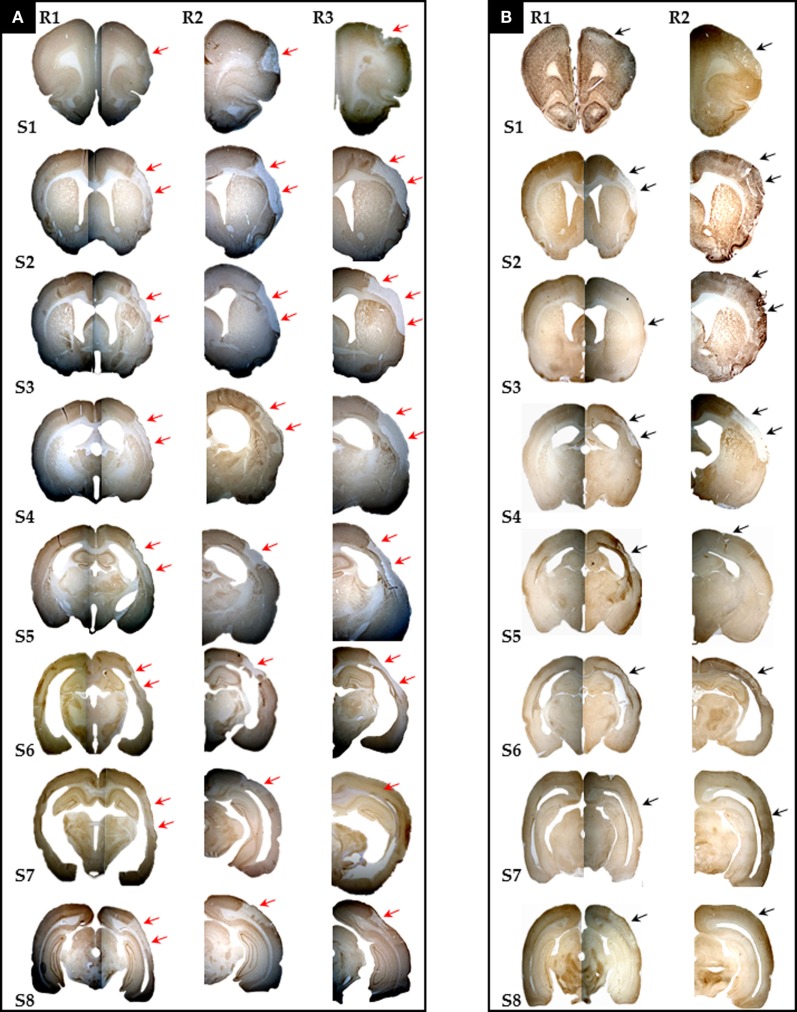
**Photomicrographs (10×) of eight coronal brain sections stained with NeuN and selected for the sake of illustration to span the whole MCA territory (S1–8 = Bregma +2.70 mm, +1.00 mm, -0.26 mm, -0.92 mm, -2.12 mm, -3.14 mm, -4.52 mm, and - 6.04 mm, respectively) from all three rats (R1 to R3) with 30 min MCAo [(A), *n* = 3] and two rats (R1 and R2) subjected to 22 min [(B), *n* = 2] MCAo, showing the presence of infarction or partial infarction in each subject (arrows)**. Pan-necrosis affected the whole range of sections in 30 min MCAo rats, while partial infarction affected the majority of sections in 22 min MCAo rats (see Table 1 for details). Selective neuronal loss was also present in all rats (see Table 1); however, this is not always obvious here due to the small magnification (see Figure 2 for details).

**Figure 2:**
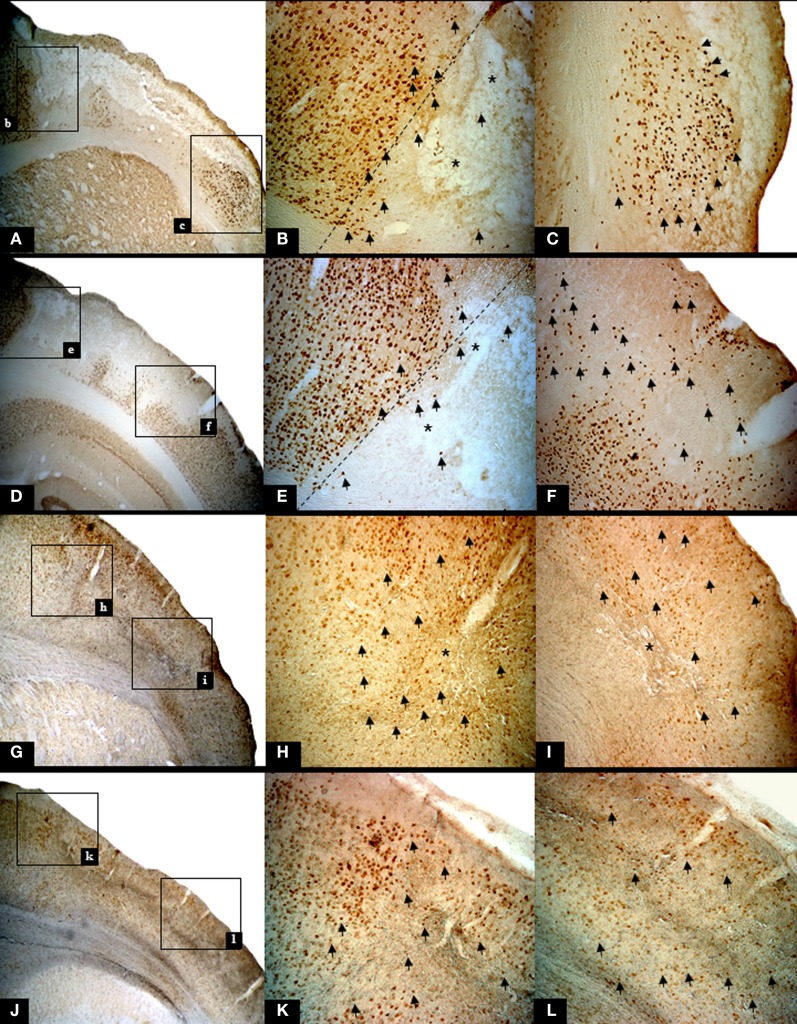
**Illustrative high resolution images of the ischemic pathology found in rats subjected to 30 min and 22 min MCAo, using NeuN staining**. The top row and row 2 show findings in one rat subjected to 30 min MCAo (Rat 2), while row 3 and bottom row show findings in one 22 min MCAo rat (Rat 2). Each left-hand image shows the area of interest at 25×, and subsequent images in the row are higher magnification (50×) of the two boxes shown in the lower magnification image. **(A)** *Infarction* (bregma +1.00 mm) with marginal area of selective neuronal loss (SNL). The following features are highlighted: nuclear pyknosis [arrows; **(B,C)**], cavitations [*; **(B)**] together with complete loss of NeuN binding **(A–C)** and cortical thinning; (**D**) *Partial infarction* (bregma -6.04 mm). The following features are highlighted: confluent areas of complete loss of staining, representing extensive neuronal death and nuclear pyknosis [arrows; **(E,F)**] with fewer cavitations [*; **(E)**] in the core, with a peripheral rim of SNL. Note that the extracellular matrix shows deteriorated cytoarchitecture. **(G)** *Partial infarction* in a rat subjected to 22 min MCAo (bregma +1.00 mm). Confluent areas of karyolysis, karyorrhexis, pyknosis [arrows; **(H,I)**], and some areas of SNL with cavitations (*) in the core. **(J)** Light microscopic histological analysis of brain sections from the same rat highlighting areas of SNL (bregma -6.04 mm). Note the presence of nuclear pyknosis [arrows; **(K,L)**].

The presence of pan-necrosis in each of the three 30 min rats, and of partial infarction in both the three 30-min and the two 22 min rats, is documented in Figure 1. Figure 2 shows higher magnification brain sections illustrating NeuN appearances for pan-necrosis and partial infarction in a 30-min rat, and partial infarction with some SNL in a 22 min MCAo rat.

**Figure 3:**
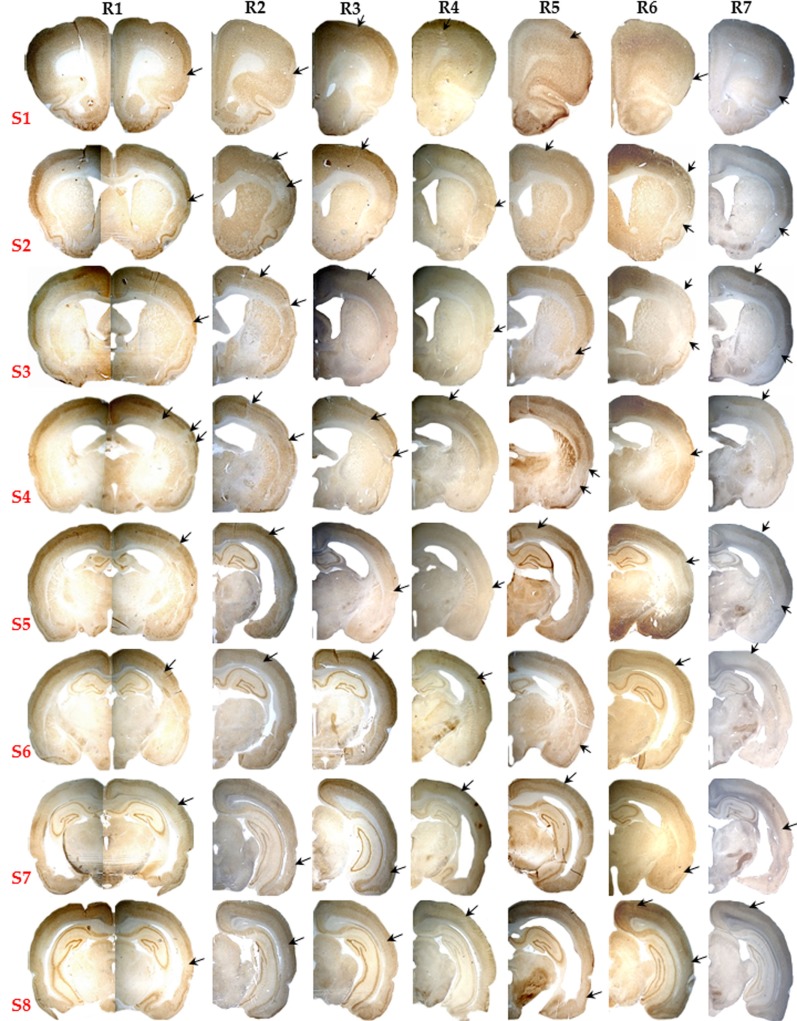
**Pictorial presentation (10×) of NeuN staining for the eight selected brain sections (S1 to S8) of all seven rats subjected to 15 min MCAo (R1 to R7)**. Note the striking absence of infarction or partial infarction in any subject. Patchy areas of pure cortical selective neuronal loss (SNL) were present in each rat, and are pointed here by arrows even though they are not always easy to identify at this low magnification (see Figure 4 for details).

**Figure 4:**
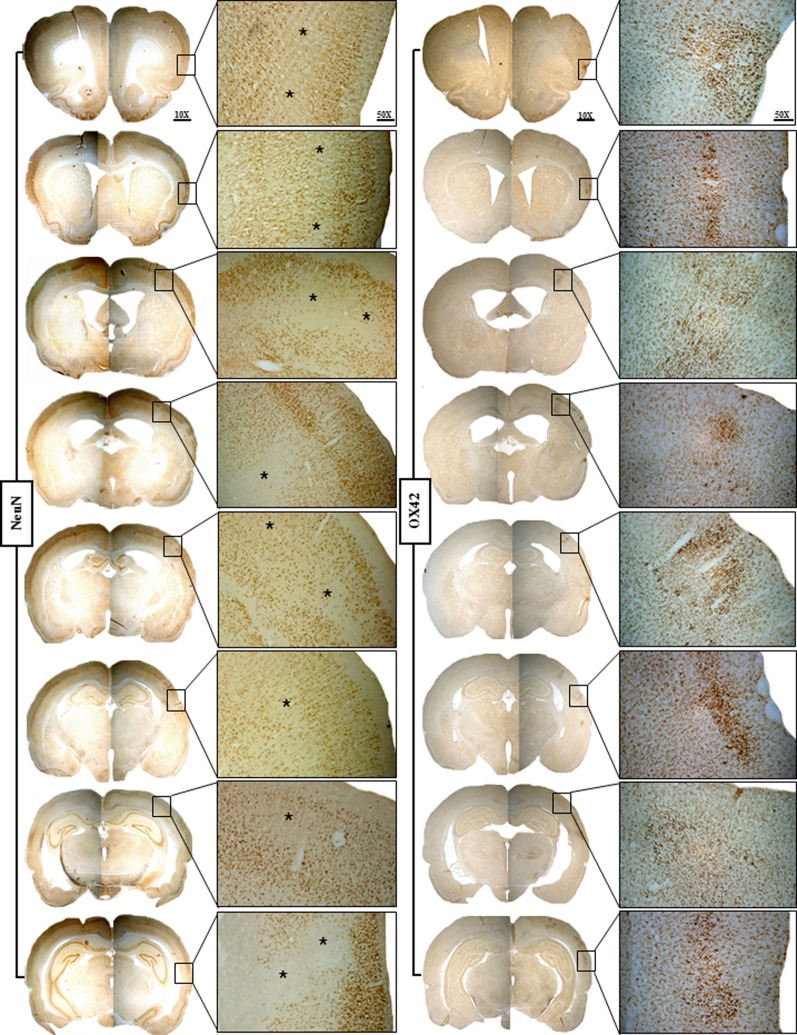
**Representative brain sections of Rat 1 of the 15 min MCAo group showing NeuN staining on the left and OX42 staining on the right for the eight selected coronal sections**. The findings are illustrated at ×25 magnification of the boxes shown at ×10 magnification. This figure highlights the striking topographical congruence of NeuN and OX42 changes in the affected cortical areas. Karyolysis, karyorrhexis, nuclear pyknosis, and patches of selective neuronal loss (SNL; *) are evident on NeuN, while increased population of highly activated microglia matching the areas of SNL is evident on OX42, an association typical for recent SNL.

**Figure 5:**
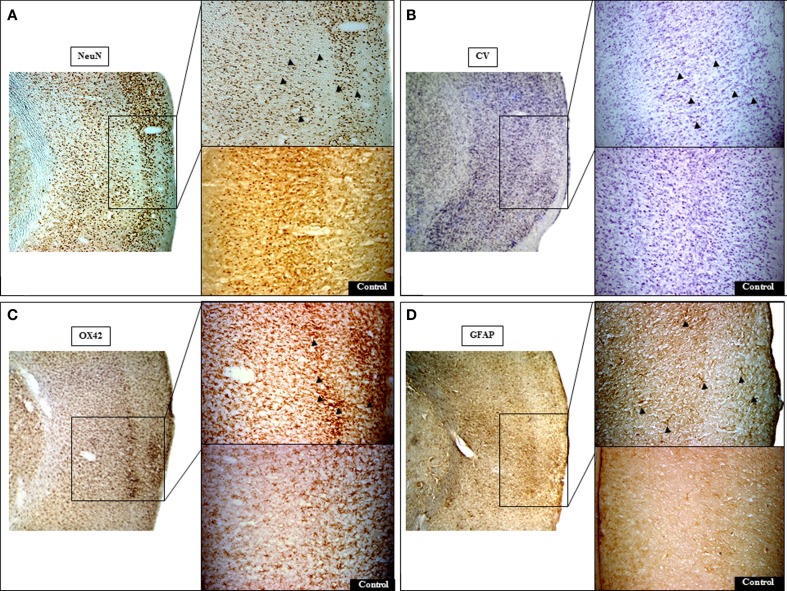
**Representative histological micrographs demonstrating pure selective neuronal loss (SNL) in 15 min MCAo rat # 1 (bregma +1 mm)**. Areas of SNL are clearly documented on NeuN **(A)** and less clearly so on Cresyl violet (CV) staining **(B)**. At higher resolution, NeuN staining revealed neuronal alterations ranging from clumping and condensation of nuclear chromatin (fragmented appearance) to pyknotic nuclei (arrowheads) that are less conspicuous on CV. Note prominent activated microglia [arrowheads; **(C)**] and astrocytosis [arrowheads; **(D)**] on OX42 and GFAP stains, respectively, topographically congruent with areas of SNL on NeuN. The contralateral cortical region (control side) exhibits normal appearances. The left image of each section represents identical sections with different staining at low magnification (25×). For each stain, the top right image shows the area of interest at higher magnification (50×).

The lack of partial/complete infarction and the presence of SNL in each of the seven 15 min rats is documented in Figure 3. SNL is clearly visible in some rats (# 1, 2, 3, and 6), and less so in the remaining rats at this low magnification. Figure 4 illustrates at high magnification in one representative rat the striking co-localization of neuronal loss, assessed with NeuN, and microglial activation, assessed using OX42. This observation was true for each 15 min MCAo rat. Figure 5 shows, from one representative rat, the findings at high magnification for all four stains used in the present study, as compared to the unaffected hemisphere. This figure illustrates firstly that the patches affected by SNL also displayed intense activated microglial as well as astrocytosis, and secondly that Cresyl violet was as expected less sensitive in depicting areas of neuronal loss as compared to NeuN.

### MRI

**Figure 6:**
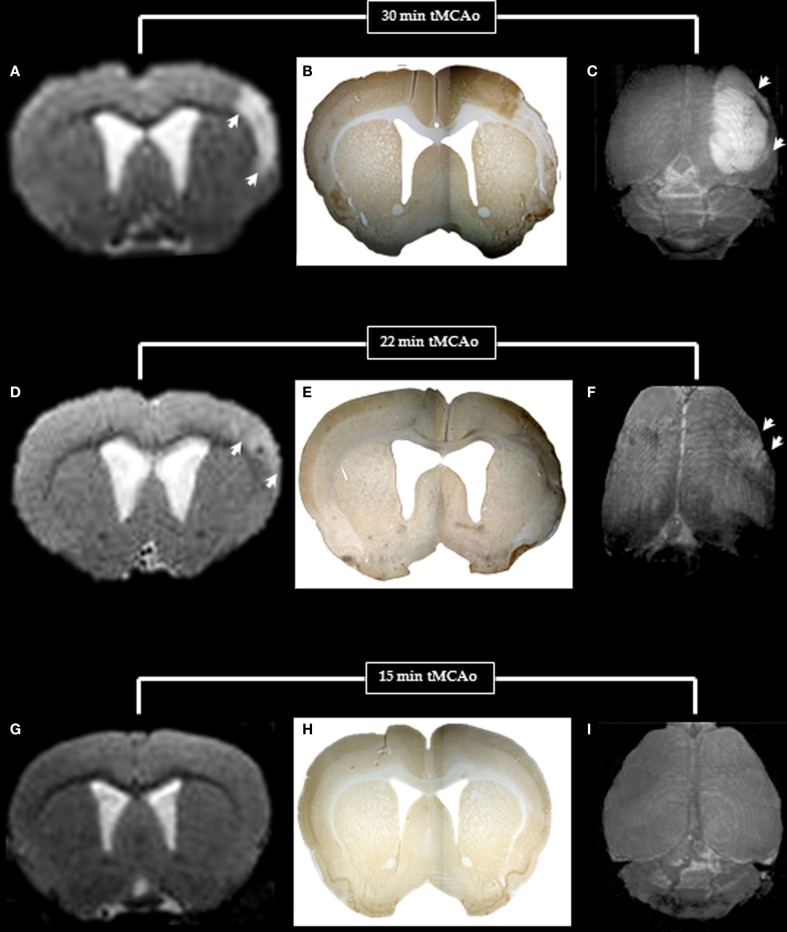
**T2-weighted MRI (left) and NeuN immunohistochemistry (middle) for one coronal section (bregma +1.00 mm), and surface rendered T2-weighted images (right) in typical rats with 30, 22, and 15 min MCAo to illustrate the MRI and histopathological counterparts of pan-necrosis (A–C), partial infarction (D–F), and pure selective neuronal loss (G–I)**. Note the excellent topographical correlation between the MRI changes and the loss of NeuN binding, whereas the surface-rendered T2-weighted images clearly demonstrate the difference in extent of damage (arrows) for the three MCAo durations.

MRA was normal in all three rats that underwent it. Table 1 summarizes the T2-weighted MRI findings. High intensity signal typical of infarction was present in the affected hemisphere in all 30 min MCAo rats, in topographical congruence with the histopathological infarcts. In 22 min rats, there were also high signal changes in the affected areas, but the signal was less intense than in the 30 min group, but again topographically congruent with the post-mortem lesions. In contrast, no abnormalities were seen in any of the 15 min rats. Figure 6 illustrates in one representative rat of each group the topographical correlation between areas of infarction/partial infarction on NeuN immunohistochemistry and high T2 signal in the 30 and 22 min MCAo rats, but pure SNL with normal MRI in the 15 min rat. On the right hand side column, surface renderings of the T2 changes are presented as 3D illustration of the lesions.

## Discussion

Using both long post-injury survival (28 days) and gold standard immunohistochemistry, we found that 15 min distal tMCAo in SHRs consistently resulted in pure cortical SNL, whereas 22 and 30 min md-tMCAo induced partial infarction and pan-necrosis, respectively. Importantly, no 15 min MCAo animals had any partial infarction or pan-necrosis and all had at least a degree of SNL, indicating consistently pure cortical SNL. Accordingly, chronic-stage T2-weighted MRI showed no T2-weighted lesion in any 15 min MCAo rat, in contrast to definite hyperintense lesions congruent with the histopathological lesion in all 22 and 30 min rats. A detailed account of the behavioral correlates of pure SNL in another sample of 15 min md-tMCAo SHRs is reported elsewhere (22). Briefly, there were no changes on the Neuroscore but significant and long-lasting deficits using subtle sensorimotor tests.

### Histopathological findings

Based on classic histopathological definitions (11, 27), we differentiated partial from complete infarction. However, it is increasingly recognized that both are in fact representations of pan-necrosis although at a less mature stage for the former (28). Thus in what follows both will be considered to reflect pan-necrosis.

As reviewed in detail elsewhere (6), scattered cortical SNL has been frequently reported in association with striatal infarction following *proximal* tMCAo, but never in isolation because the striato-capsular area is known to sustain the brunt of the ischemic insult following proximal MCAo. In other words, proximal MCAo is not a candidate model for pure cortical SNL.

Apart from our group (18, 19), *distal* tMCAo in rats has been used in four previous studies only (16, 17, 29, 30). However, in all three studies using SHRs, the shortest MCAo duration was 1 h, and not unexpectedly infarction was consistently reported (16, 17, 29). Interestingly, two of the above studies (16, 17) also mention the presence of “scattered cortical neuronal death,”, both assessed with H&E only, likely representing SNL but this is difficult to ensure due to the lack of specific neuronal markers (6). Implementing 1 h distal tMCAo in Wistar rats, Qiao et al. (30) similarly found partial or complete infarcts; however, in a group of 10 rats subjected to 40 min tMCAo, they reported the presence at day 7 of mild cortical SNL together with astrocytosis and mild MA and normal T2-weighted MRI. However, no further details on these findings is provided and only H&E was used. Furthermore, whether SNL consistently affected each of their 40 min tMCAo rats is not stated, and MCAo duration was not systematically varied as we did here. Despite these caveats, however, the results from this previous study (30) appear consistent with ours. That a 40 min MCAo duration may result in pure SNL outcome in Wistar rats as compared to 15 min in SHR is consistent with the well-established greater sensitivity to ischemia in the latter (31) due to poorer pial collaterals (32). Accordingly, in our previous set of studies using 45 min md-tMCAo in SHRs, the majority of subjects exhibited infarction (18, 26).

In the present study, we harvested brains 28 days after the ischemic insult. This long interval was deliberately chosen in order to ensure full maturation of the ischemic lesions. All studies that have assessed the time-course of histopathological changes following tMCAo in rodents point to rapidly evolving infarct maturation dependent of occlusion time and length of re-perfusion, and consistently complete well within 7 days (11, 33–35). However, based on observations of apparently delayed infarct maturation, Lehrmann et al. stated that “The longer the ischemic duration, the faster the neuronal damage evolved from ischemic or homogenized cell damage to neuronal loss with gliosis” (15). A similar phenomenon has been reported in two additional literature reports (36, 37). Although these observations were necessarily based on separate small samples of rats and are therefore of uncertain significance, we elected to sit on the safe side in the present investigation and opted for a long survival delay, namely 28 days.

### MRI findings

T2 hyperintense lesions are the MRI hallmark of infarction in both the experimental and clinical settings (29, 38–40). Consistent with previous studies (18, 41), the MR counterpart of partial infarction was again a definite, albeit less conspicuous T2 hyperintense signal. In contrast, cortical SNL was not associated with any T2-weighted abnormalities, consistent with previous reports of either striatal or cortical SNL (10, 13, 14, 18, 30).

## Conflict of Interest Statement

The authors declare that the research was conducted in the absence of any commercial or financial relationships that could be construed as a potential conflict of interest.
